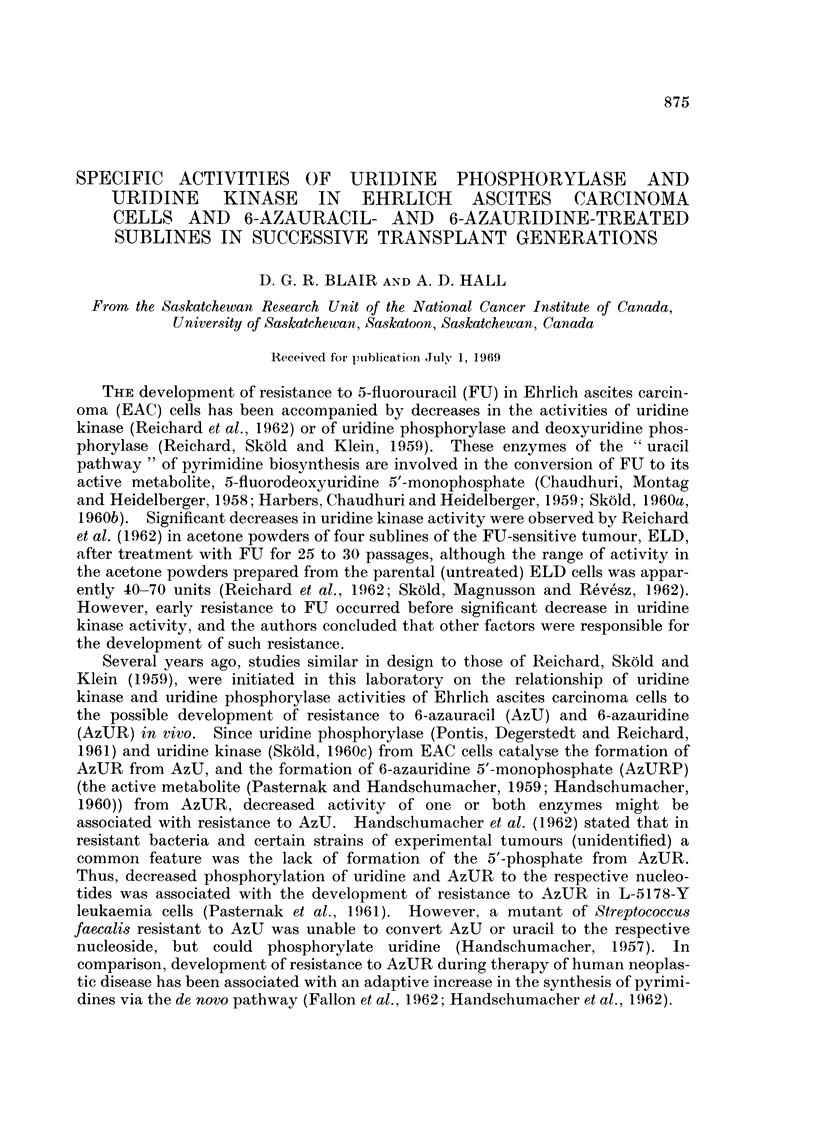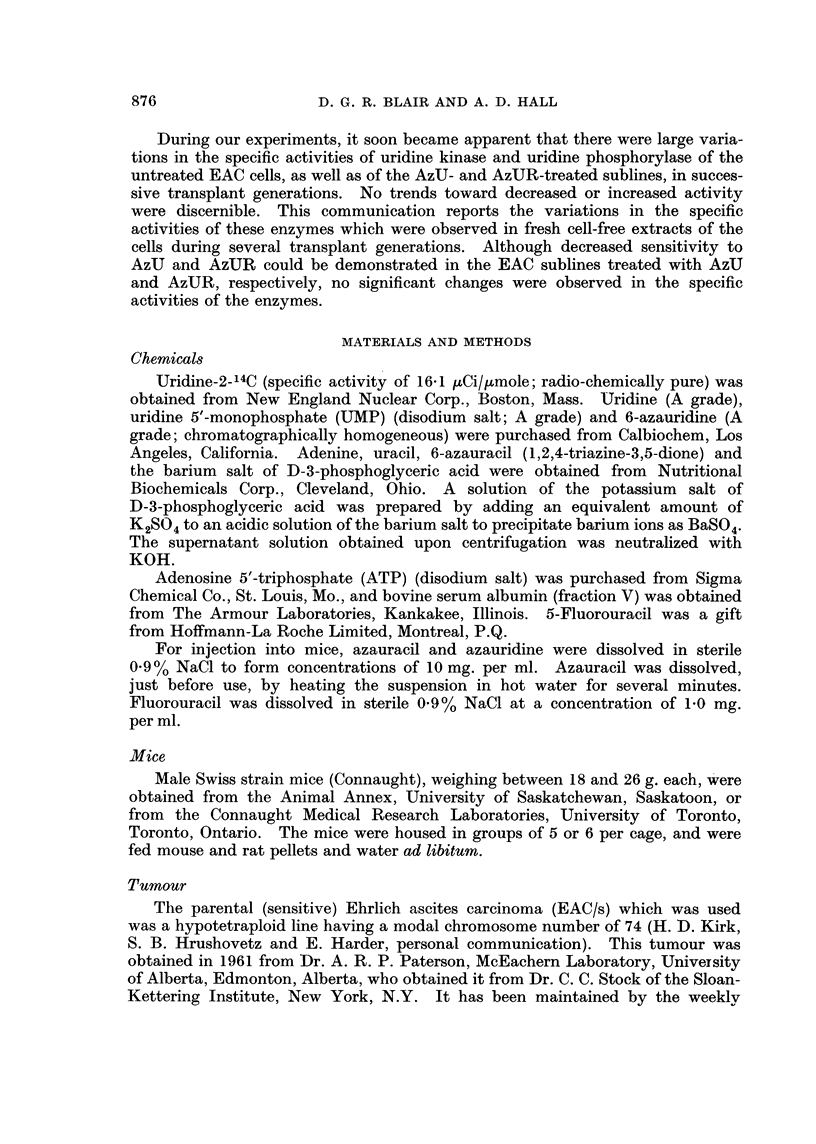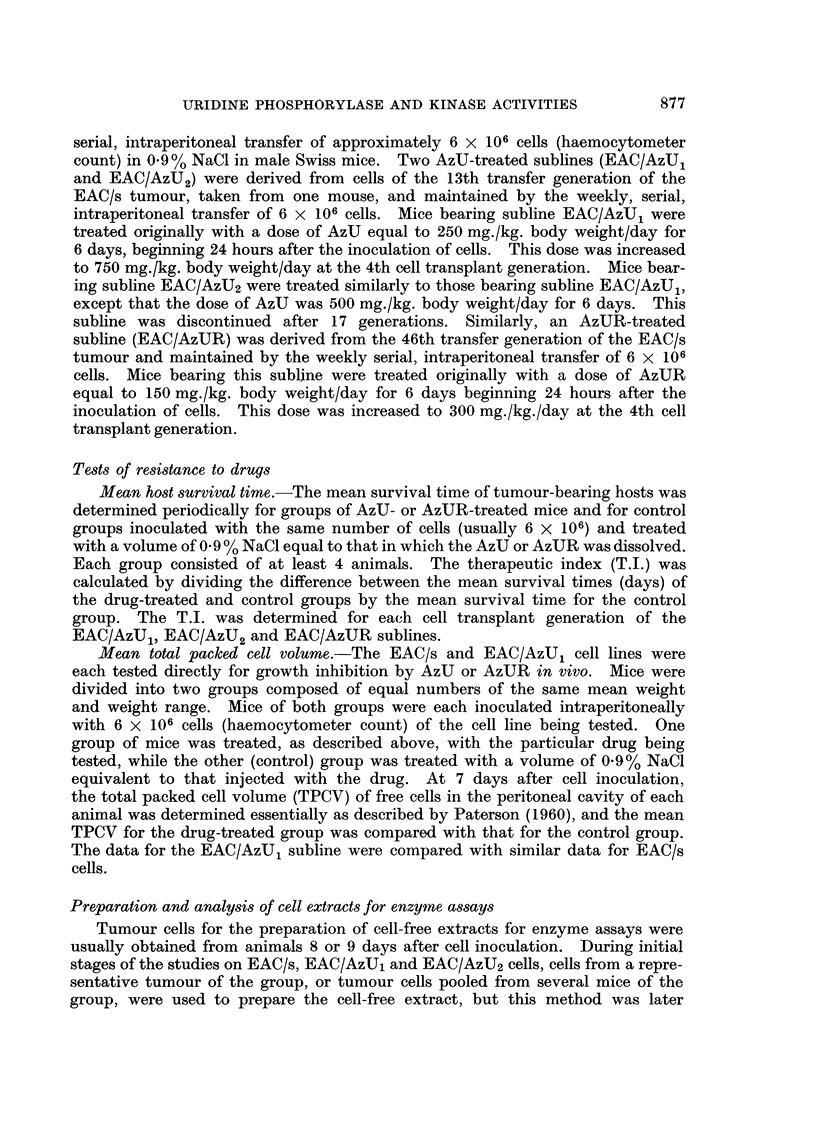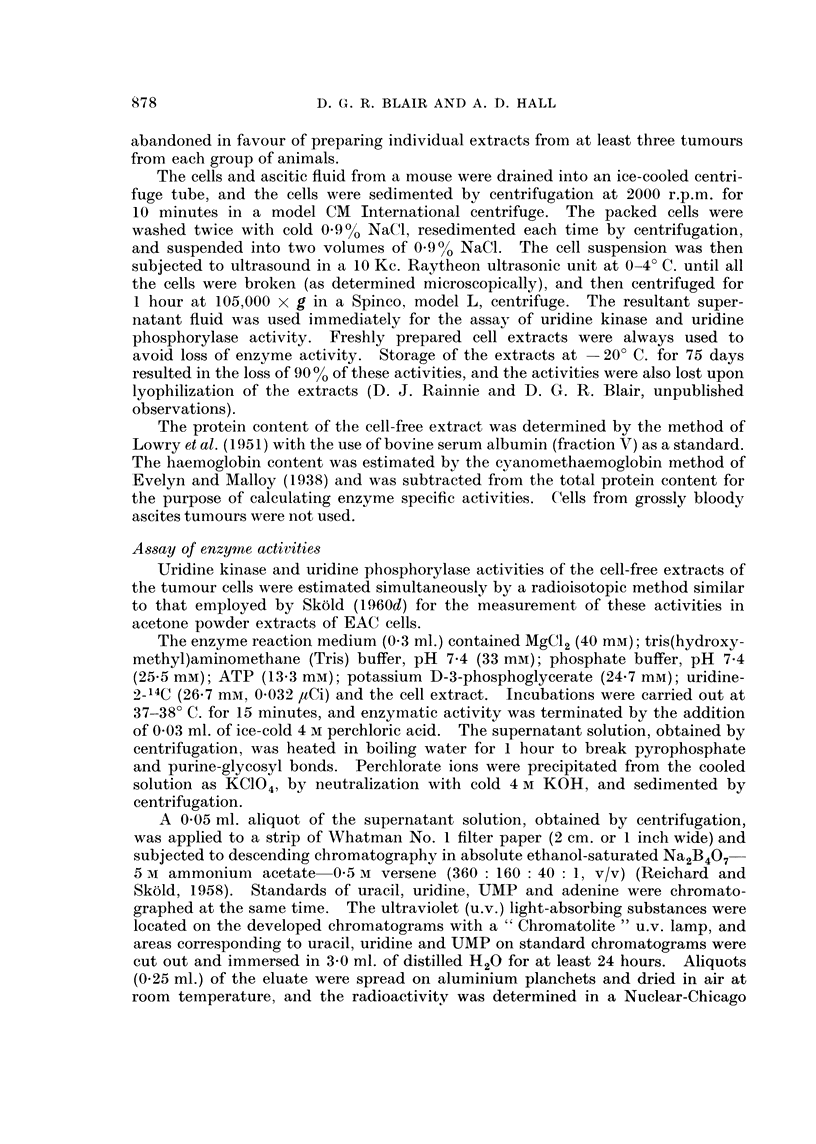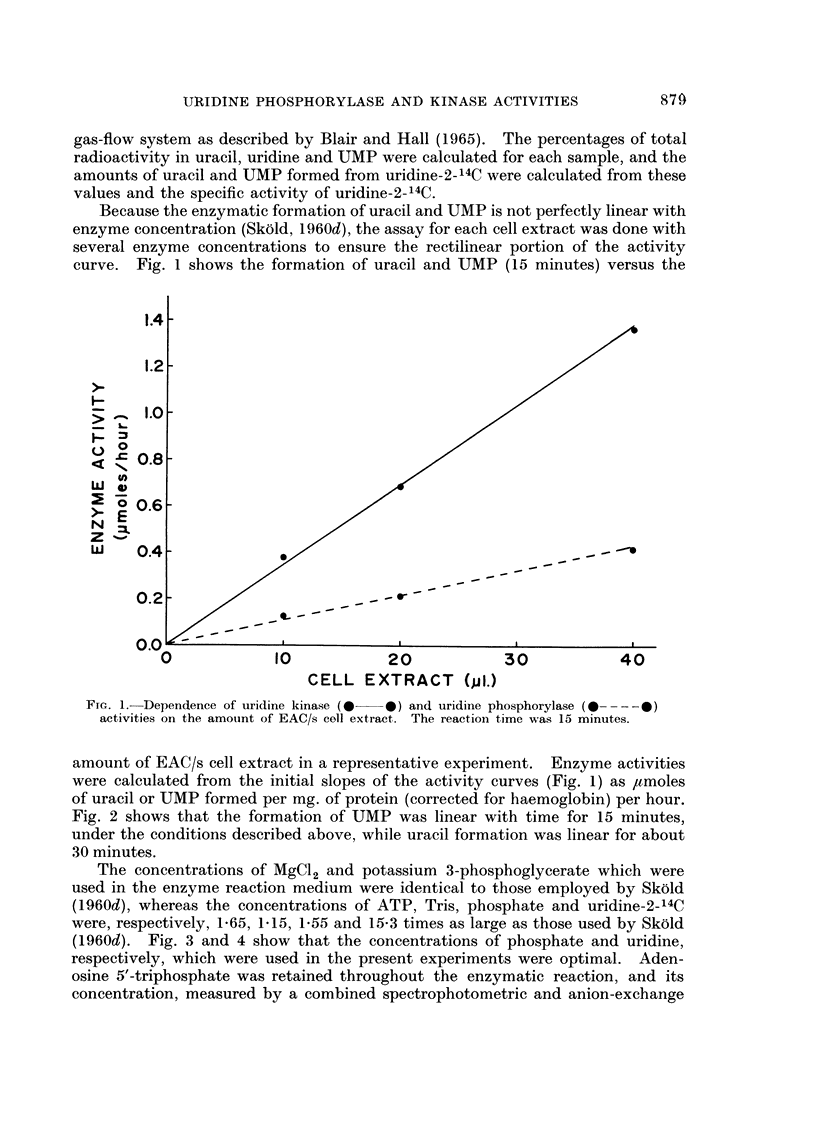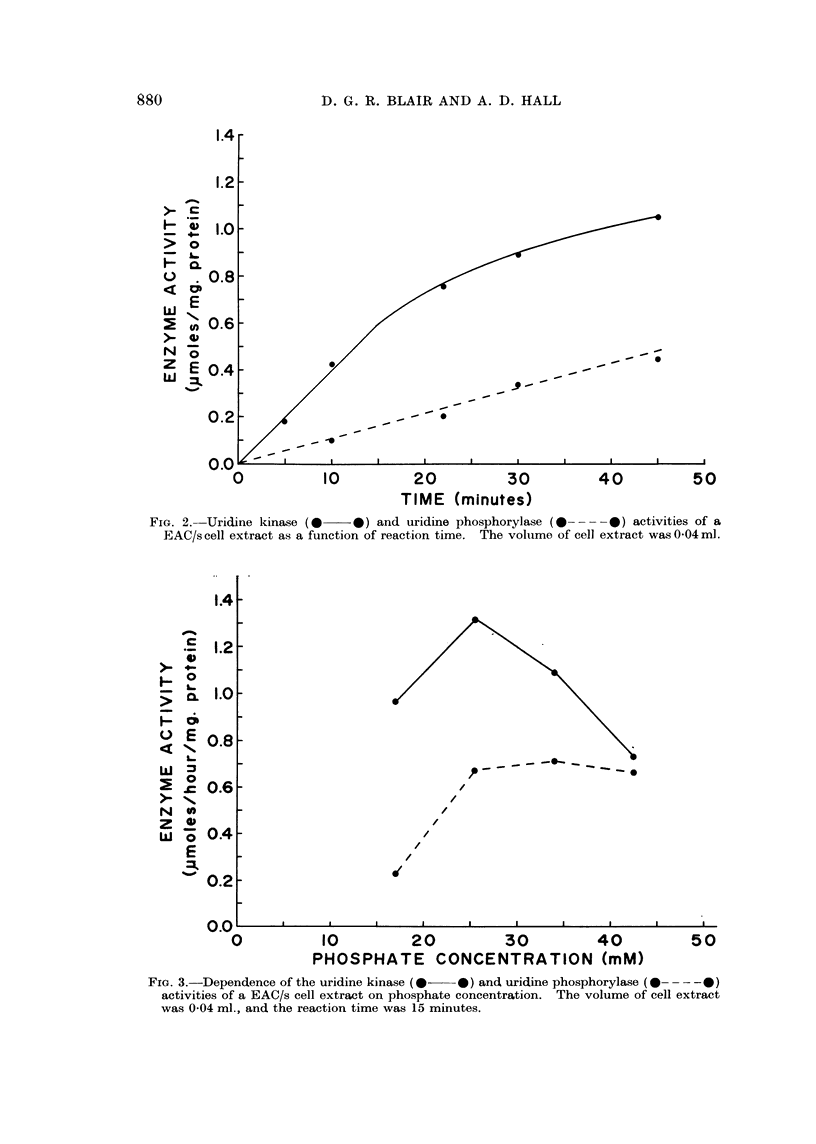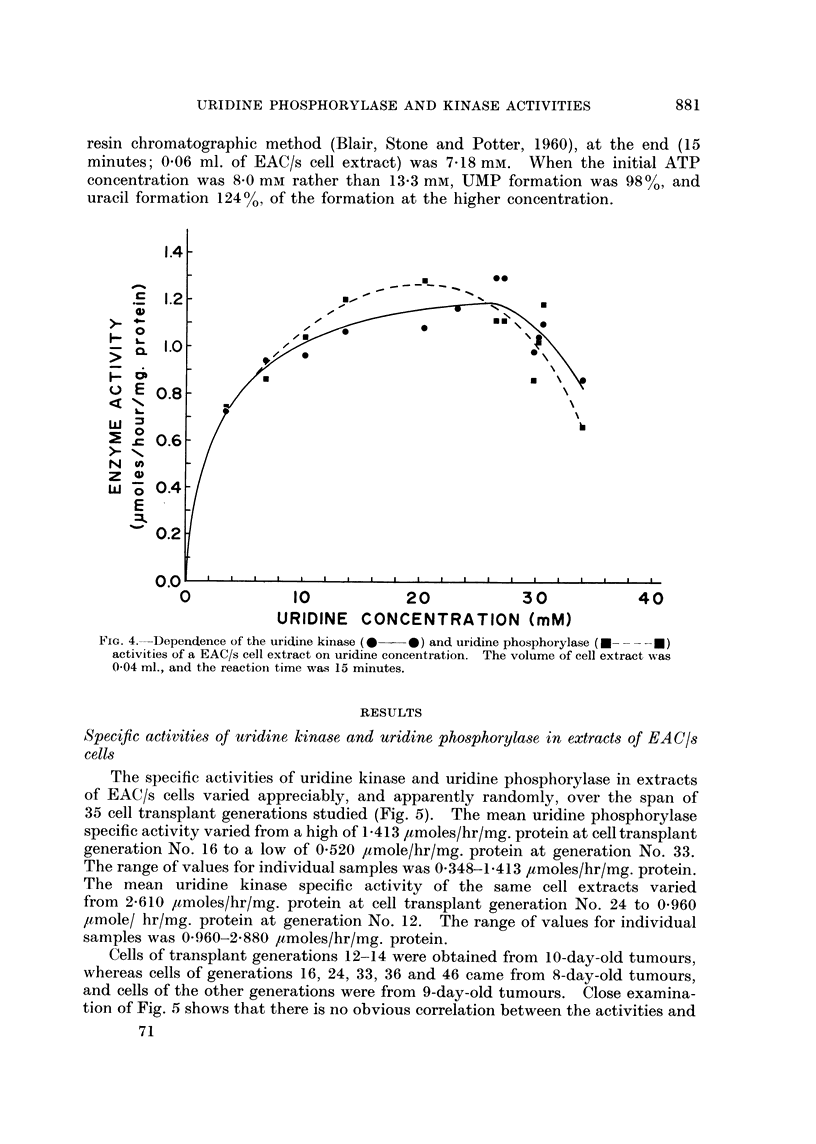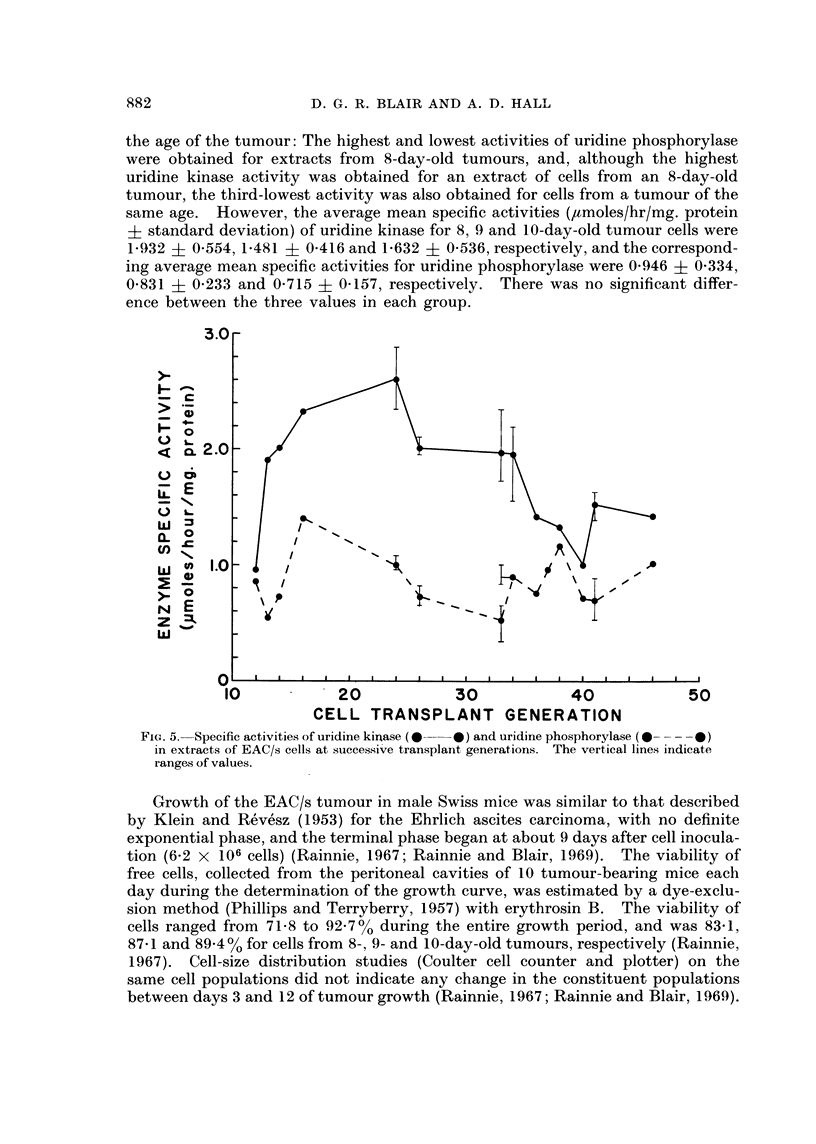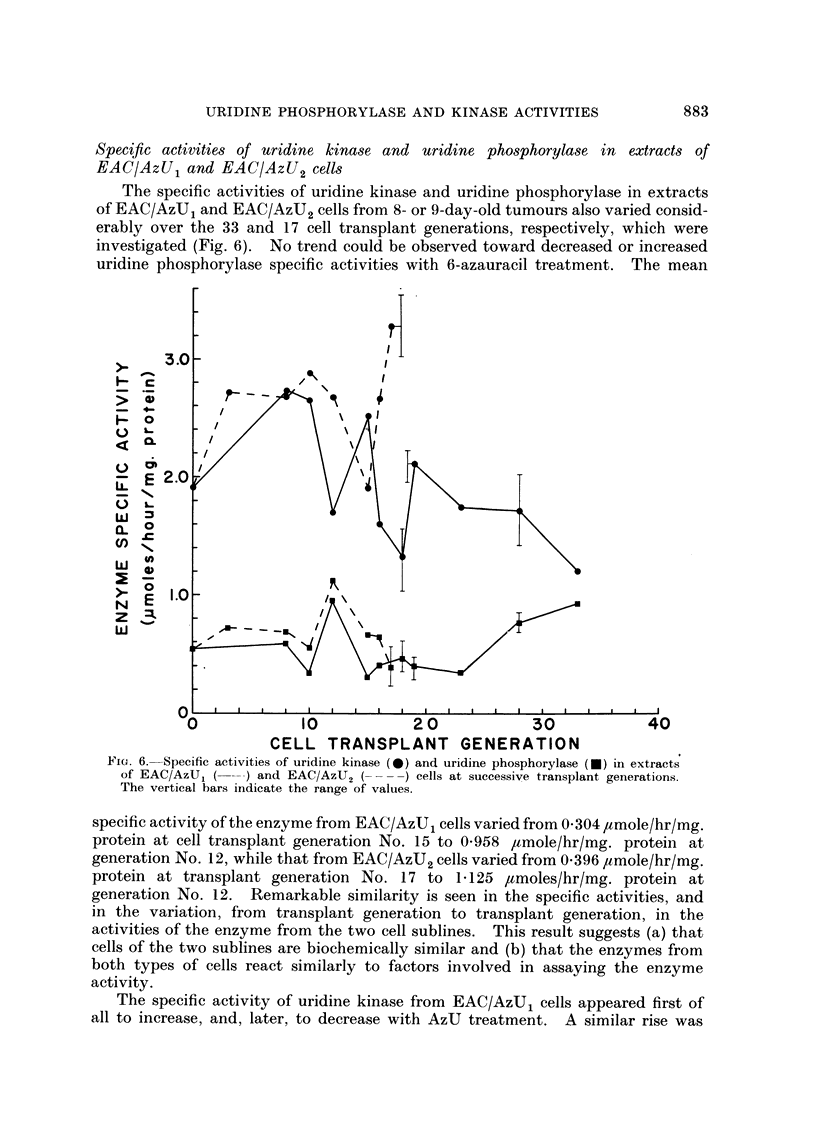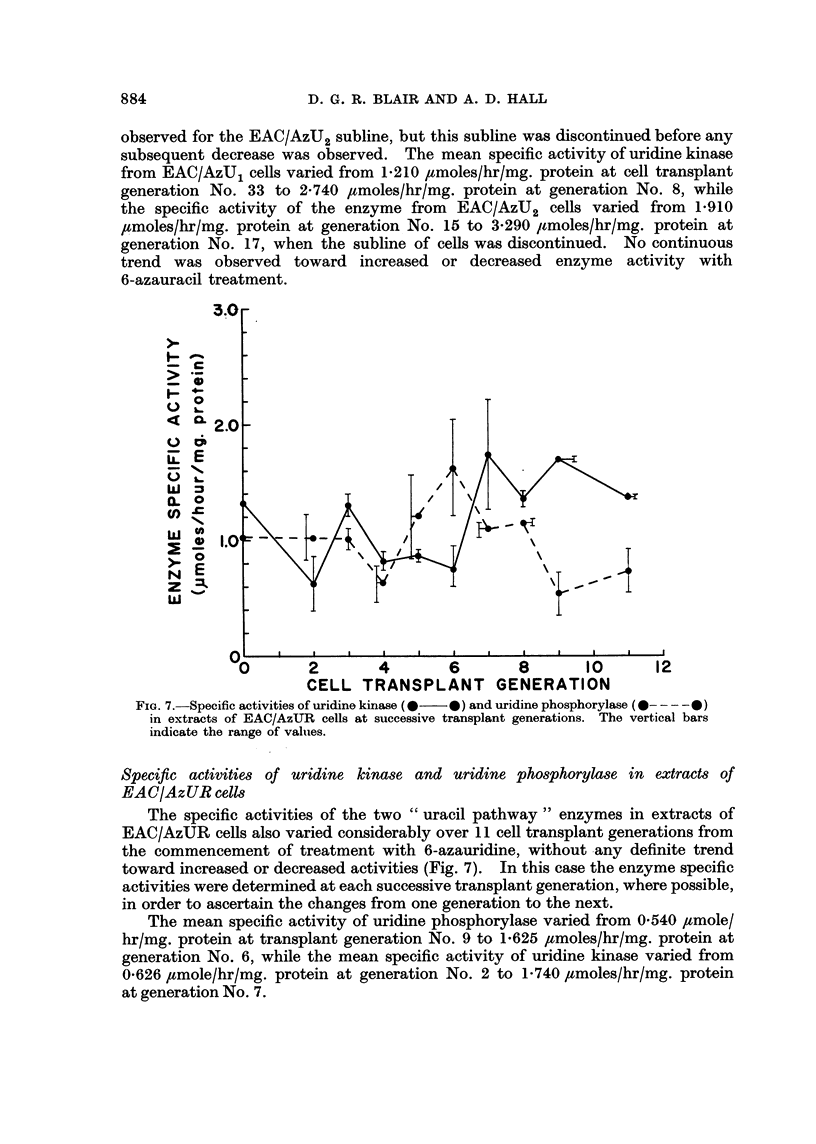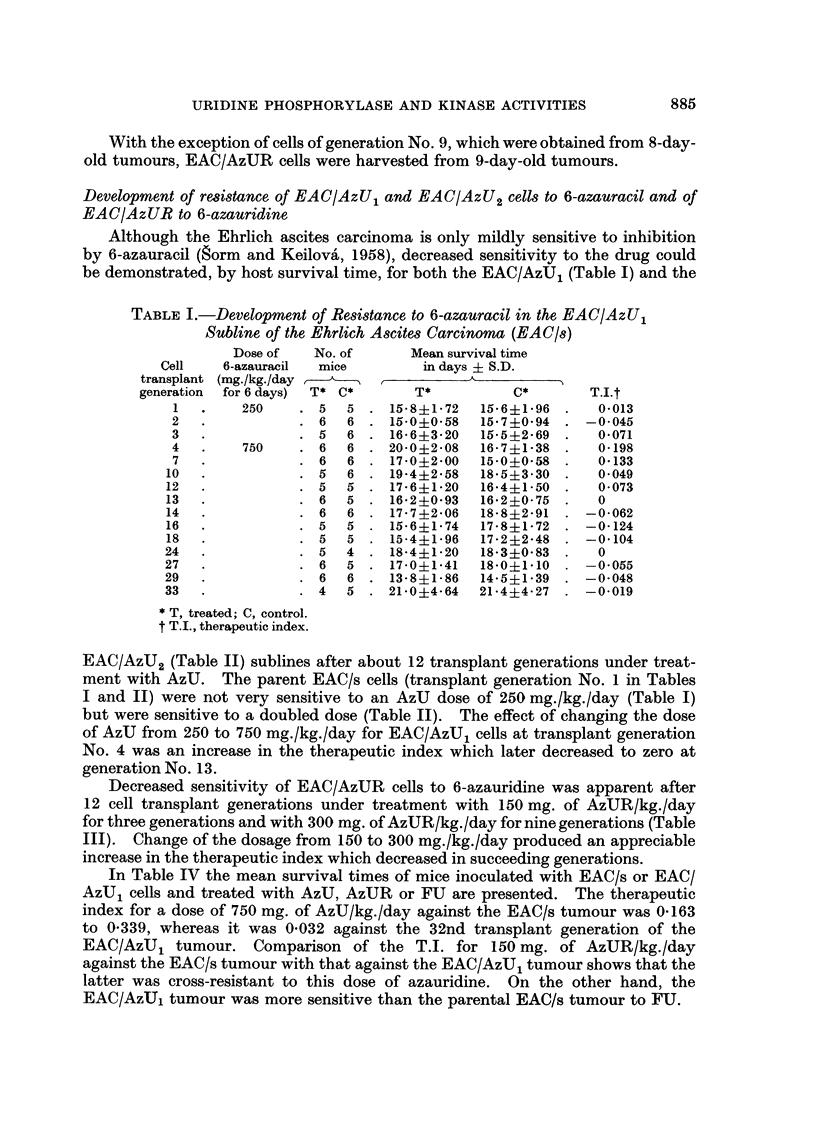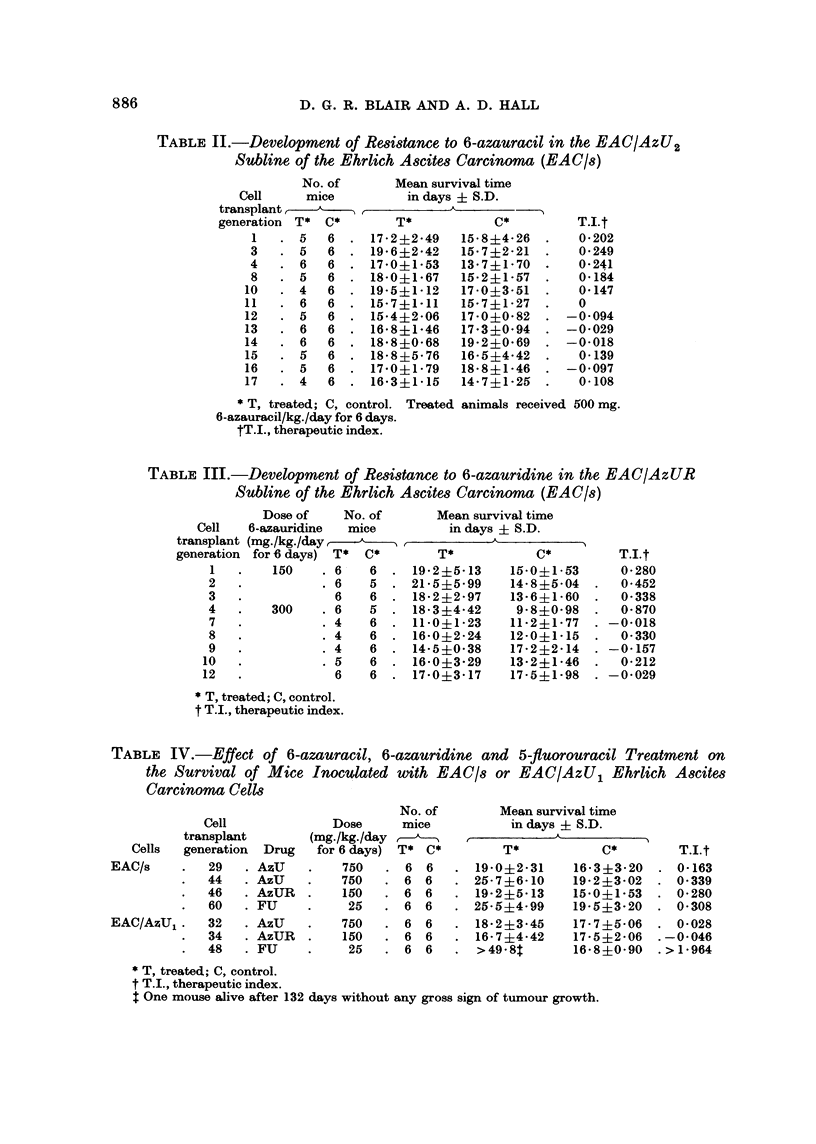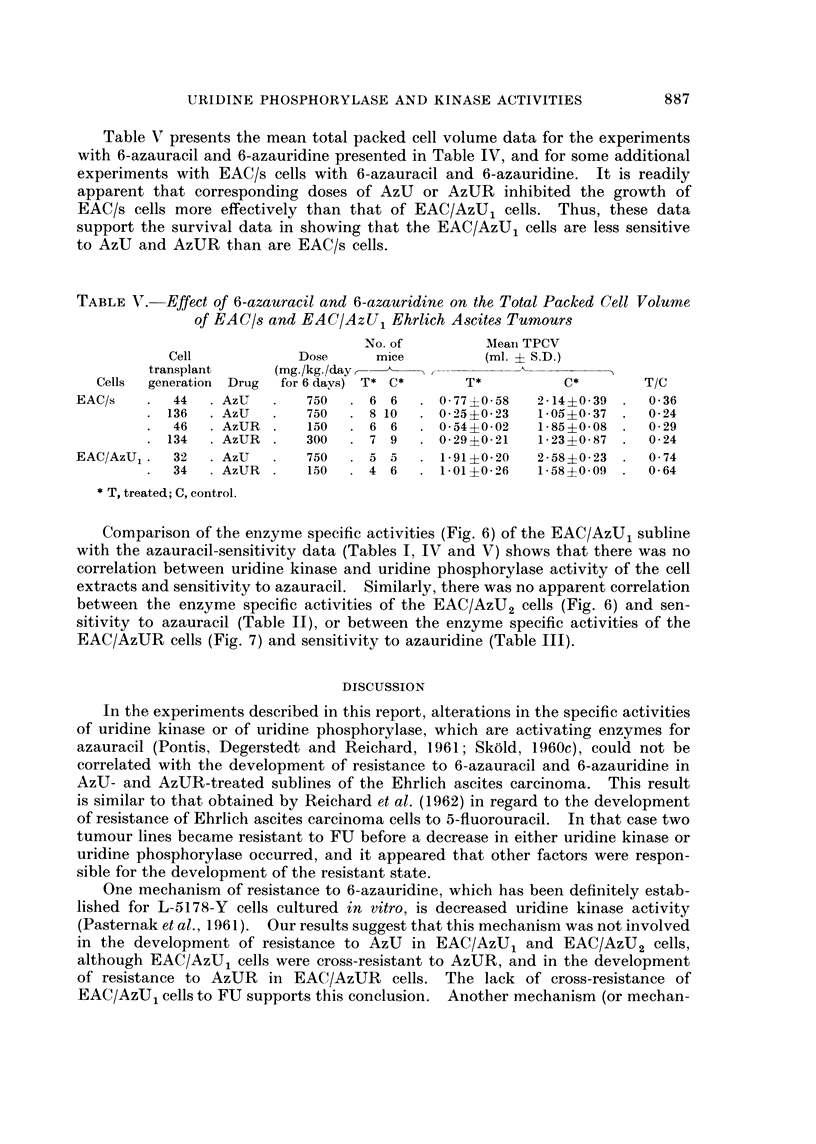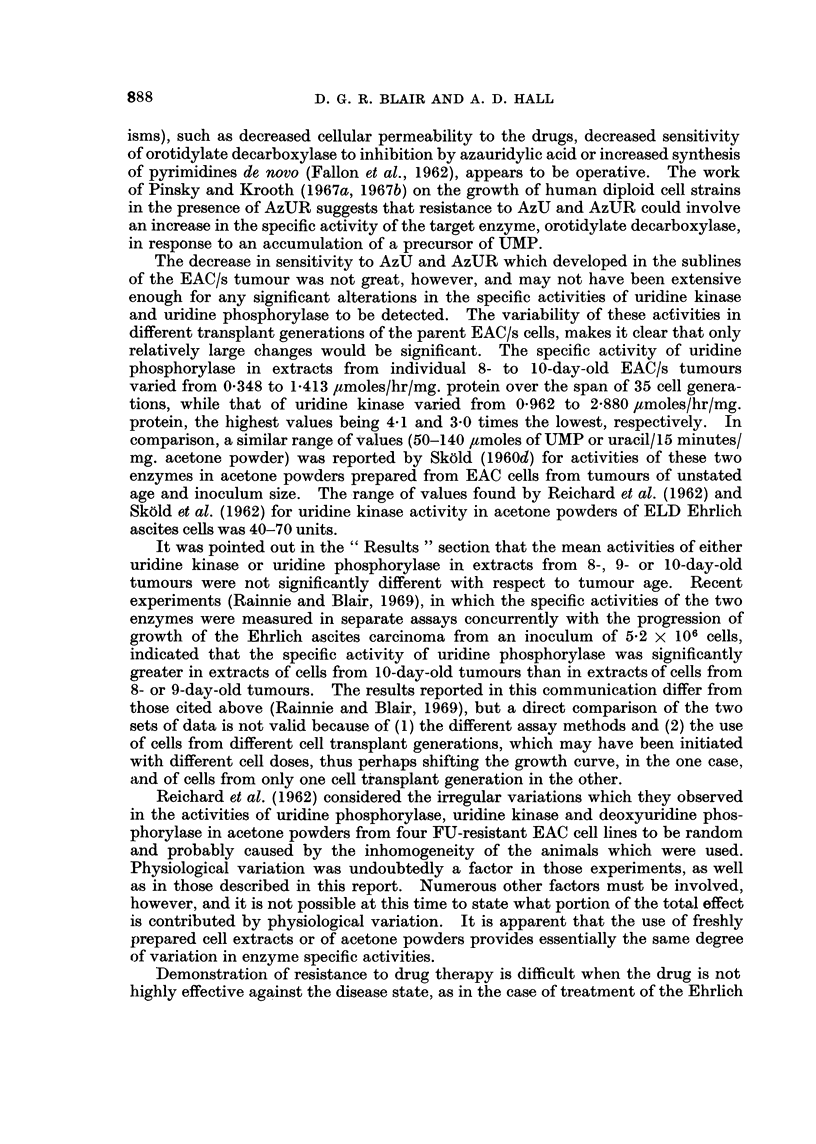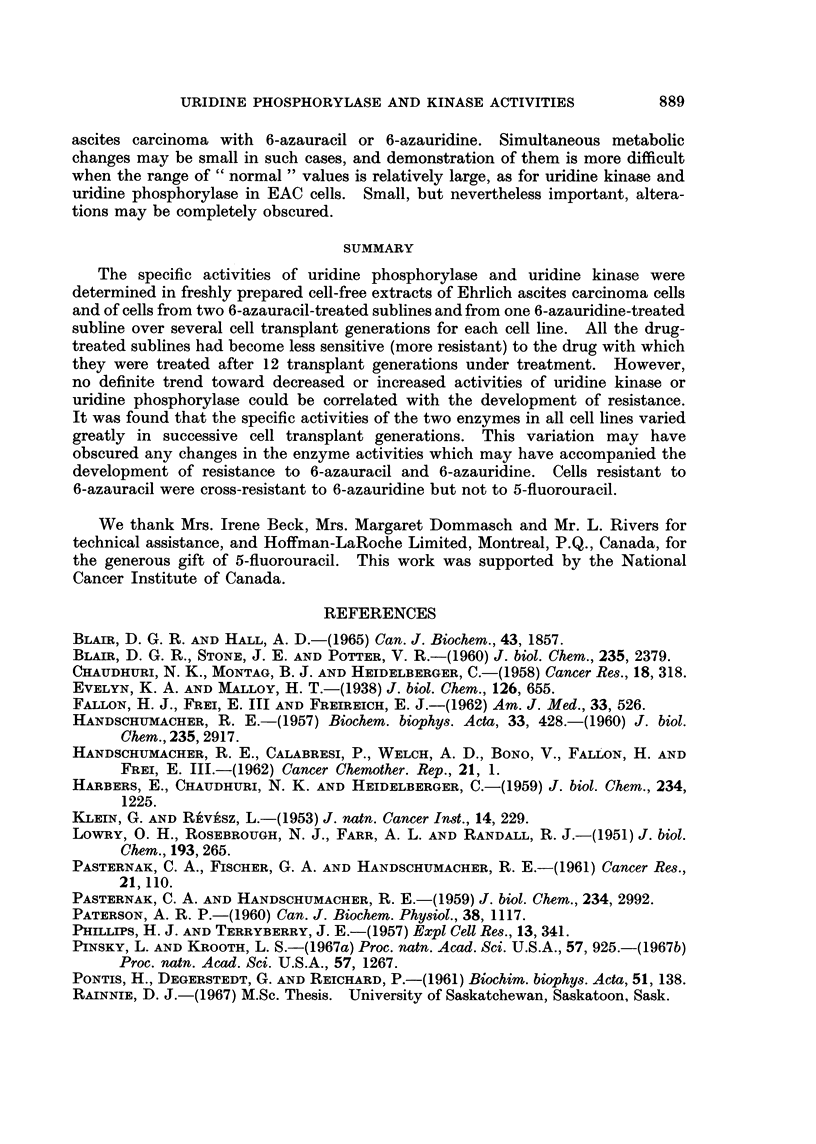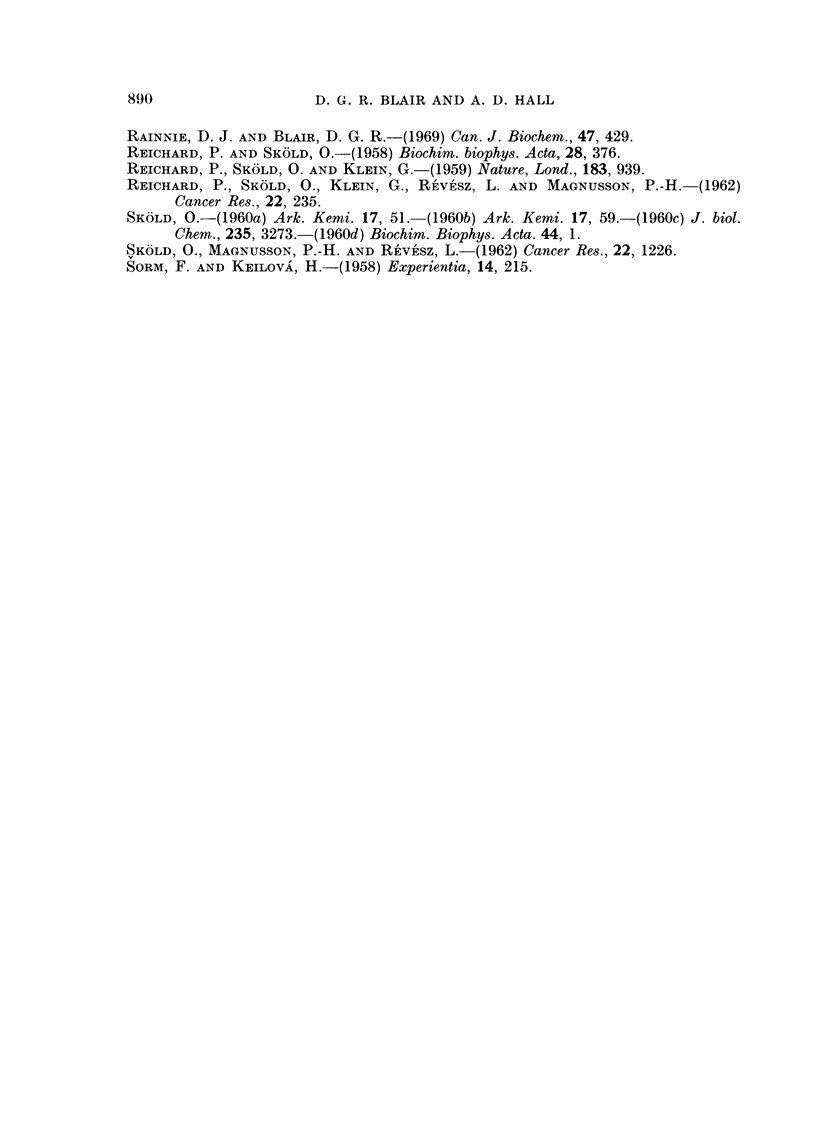# Specific activities of uridine phosphorylase and uridine kinase in Ehrlich ascites carcinoma cells and 6-azauracil- and 6-azauridine-treated sublines in successive transplant generations.

**Published:** 1969-12

**Authors:** D. G. Blair, A. D. Hall


### SPECIFIC ACTIVITIES OF URIDINE PHOSPHORYLASE AND URIDINE KINASE IN EHRLICH ASCITES CARCINOMA CELLS AND 6-AZAURACIL- AND 6-AZAURIDINE-TREATED SUBLINES IN SUCCESSIVE TRANSPLANT GENERATIONS

D. G. R. BLAIR AND A. D. HALL

*From the Saskatchewan Research Unit of the National Cancer Institute of Canada, University of Saskatchewan, Saskatoon, Saskatchewan, Canada*

Received for publication July 1, 1969

THE development of resistance to 5-fluorouracil (FU) in Ehrlich ascites carcinoma (EAC) cells has been accompanied by decreases in the activities of uridine kinase (Reichard *et al.*, 1962) or of uridine phosphorylase and deoxyuridine phosphorylase (Reichard, Sköld and Klein, 1959). These enzymes of the " uracil pathway " of pyrimidine biosynthesis are involved in the conversion of FU to its active metabolite, 5-fluorodeoxyuridine 5′-monophosphate (Chaudhuri, Montag and Heidelberger, 1958; Harbers, Chaudhuri and Heidelberger, 1959; Sköld, 1960*a*, 1960*b*). Significant decreases in uridine kinase activity were observed by Reichard *et al.* (1962) in acetone powders of four sublines of the FU-sensitive tumour, ELD, after treatment with FU for 25 to 30 passages, although the range of activity in the acetone powders prepared from the parental (untreated) ELD cells was apparently 40–70 units (Reichard *et al.*, 1962; Sköld, Magnusson and Révész, 1962). However, early resistance to FU occurred before significant decrease in uridine kinase activity, and the authors concluded that other factors were responsible for the development of such resistance.

Several years ago, studies similar in design to those of Reichard, Sköld and Klein (1959), were initiated in this laboratory on the relationship of uridine kinase and uridine phosphorylase activities of Ehrlich ascites carcinoma cells to the possible development of resistance to 6-azauracil (AzU) and 6-azauridine (AzUR) *in vivo*. Since uridine phosphorylase (Pontis, Degerstedt and Reichard, 1961) and uridine kinase (Sköld, 1960*c*) from EAC cells catalyse the formation of AzUR from AzU, and the formation of 6-azauridine 5′-monophosphate (AzURP) (the active metabolite (Pasternak and Handschumacher, 1959; Handschumacher, 1960)) from AzUR, decreased activity of one or both enzymes might be associated with resistance to AzU. Handschumacher *et al.* (1962) stated that in resistant bacteria and certain strains of experimental tumours (unidentified) a common feature was the lack of formation of the 5′-phosphate from AzUR. Thus, decreased phosphorylation of uridine and AzUR to the respective nucleotides was associated with the development of resistance to AzUR in L-5178-Y leukaemia cells (Pasternak *et al.*, 1961). However, a mutant of *Streptococcus faecalis* resistant to AzU was unable to convert AzU or uracil to the respective nucleoside, but could phosphorylate uridine (Handschumacher, 1957). In comparison, development of resistance to AzUR during therapy of human neoplastic disease has been associated with an adaptive increase in the synthesis of pyrimidines via the *de novo* pathway (Fallon *et al.*, 1962; Handschumacher *et al.*, 1962).

During our experiments, it soon became apparent that there were large variations in the specific activities of uridine kinase and uridine phosphorylase of the untreated EAC cells, as well as of the AzU- and AzUR-treated sublines, in successive transplant generations. No trends toward decreased or increased activity were discernible. This communication reports the variations in the specific activities of these enzymes which were observed in fresh cell-free extracts of the cells during several transplant generations. Although decreased sensitivity to AzU and AzUR could be demonstrated in the EAC sublines treated with AzU and AzUR, respectively, no significant changes were observed in the specific activities of the enzymes.

### MATERIALS AND METHODS

### *Chemicals*

Uridine-2-$^{14}$C (specific activity of 16·1 $\mu$Ci/$\mu$mole; radio-chemically pure) was obtained from New England Nuclear Corp., Boston, Mass. Uridine (A grade), uridine 5′-monophosphate (UMP) (disodium salt; A grade) and 6-azauridine (A grade; chromatographically homogeneous) were purchased from Calbiochem, Los Angeles, California. Adenine, uracil, 6-azauracil (1,2,4-triazine-3,5-dione) and the barium salt of D-3-phosphoglyceric acid were obtained from Nutritional Biochemicals Corp., Cleveland, Ohio. A solution of the potassium salt of D-3-phosphoglyceric acid was prepared by adding an equivalent amount of $K_2SO_4$ to an acidic solution of the barium salt to precipitate barium ions as $BaSO_4$. The supernatant solution obtained upon centrifugation was neutralized with KOH.

Adenosine 5′-triphosphate (ATP) (disodium salt) was purchased from Sigma Chemical Co., St. Louis, Mo., and bovine serum albumin (fraction V) was obtained from The Armour Laboratories, Kankakee, Illinois. 5-Fluorouracil was a gift from Hoffmann-La Roche Limited, Montreal, P.Q.

For injection into mice, azauracil and azauridine were dissolved in sterile 0·9% NaCl to form concentrations of 10 mg. per ml. Azauracil was dissolved, just before use, by heating the suspension in hot water for several minutes. Fluorouracil was dissolved in sterile 0·9% NaCl at a concentration of 1·0 mg. per ml.

### *Mice*

Male Swiss strain mice (Connaught), weighing between 18 and 26 g. each, were obtained from the Animal Annex, University of Saskatchewan, Saskatoon, or from the Connaught Medical Research Laboratories, University of Toronto, Toronto, Ontario. The mice were housed in groups of 5 or 6 per cage, and were fed mouse and rat pellets and water *ad libitum.*

### *Tumour*

The parental (sensitive) Ehrlich ascites carcinoma (EAC/s) which was used was a hypotetraploid line having a modal chromosome number of 74 (H. D. Kirk, S. B. Hrushovetz and E. Harder, personal communication). This tumour was obtained in 1961 from Dr. A. R. P. Paterson, McEachern Laboratory, University of Alberta, Edmonton, Alberta, who obtained it from Dr. C. C. Stock of the Sloan-Kettering Institute, New York, N.Y. It has been maintained by the weekly serial, intraperitoneal transfer of approximately $6 \times 10^6$ cells (haemocytometer count) in 0·9% NaCl in male Swiss mice. Two AzU-treated sublines (EAC/AzU$_1$ and EAC/AzU$_2$) were derived from cells of the 13th transfer generation of the EAC/s tumour, taken from one mouse, and maintained by the weekly, serial, intraperitoneal transfer of $6 \times 10^6$ cells. Mice bearing subline EAC/AzU$_1$ were treated originally with a dose of AzU equal to 250 mg./kg. body weight/day for 6 days, beginning 24 hours after the inoculation of cells. This dose was increased to 750 mg./kg. body weight/day at the 4th cell transplant generation. Mice bearing subline EAC/AzU$_2$ were treated similarly to those bearing subline EAC/AzU$_1$, except that the dose of AzU was 500 mg./kg. body weight/day for 6 days. This subline was discontinued after 17 generations. Similarly, an AzUR-treated subline (EAC/AzUR) was derived from the 46th transfer generation of the EAC/s tumour and maintained by the weekly serial, intraperitoneal transfer of $6 \times 10^6$ cells. Mice bearing this subline were treated originally with a dose of AzUR equal to 150 mg./kg. body weight/day for 6 days beginning 24 hours after the inoculation of cells. This dose was increased to 300 mg./kg./day at the 4th cell transplant generation.

*Tests of resistance to drugs*

*Mean host survival time.*—The mean survival time of tumour-bearing hosts was determined periodically for groups of AzU- or AzUR-treated mice and for control groups inoculated with the same number of cells (usually $6 \times 10^6$) and treated with a volume of 0·9% NaCl equal to that in which the AzU or AzUR was dissolved. Each group consisted of at least 4 animals. The therapeutic index (T.I.) was calculated by dividing the difference between the mean survival times (days) of the drug-treated and control groups by the mean survival time for the control group. The T.I. was determined for each cell transplant generation of the EAC/AzU$_1$, EAC/AzU$_2$ and EAC/AzUR sublines.

*Mean total packed cell volume.*—The EAC/s and EAC/AzU$_1$ cell lines were each tested directly for growth inhibition by AzU or AzUR *in vivo*. Mice were divided into two groups composed of equal numbers of the same mean weight and weight range. Mice of both groups were each inoculated intraperitoneally with $6 \times 10^6$ cells (haemocytometer count) of the cell line being tested. One group of mice was treated, as described above, with the particular drug being tested, while the other (control) group was treated with a volume of 0·9% NaCl equivalent to that injected with the drug. At 7 days after cell inoculation, the total packed cell volume (TPCV) of free cells in the peritoneal cavity of each animal was determined essentially as described by Paterson (1960), and the mean TPCV for the drug-treated group was compared with that for the control group. The data for the EAC/AzU$_1$ subline were compared with similar data for EAC/s cells.

*Preparation and analysis of cell extracts for enzyme assays*

Tumour cells for the preparation of cell-free extracts for enzyme assays were usually obtained from animals 8 or 9 days after cell inoculation. During initial stages of the studies on EAC/s, EAC/AzU$_1$ and EAC/AzU$_2$ cells, cells from a representative tumour of the group, or tumour cells pooled from several mice of the group, were used to prepare the cell-free extract, but this method was later abandoned in favour of preparing individual extracts from at least three tumours from each group of animals.

The cells and ascitic fluid from a mouse were drained into an ice-cooled centrifuge tube, and the cells were sedimented by centrifugation at 2000 r.p.m. for 10 minutes in a model CM International centrifuge. The packed cells were washed twice with cold 0·9% NaCl, resedimented each time by centrifugation, and suspended into two volumes of 0·9% NaCl. The cell suspension was then subjected to ultrasound in a 10 Kc. Raytheon ultrasonic unit at 0–4° C. until all the cells were broken (as determined microscopically), and then centrifuged for 1 hour at 105,000 × ***g*** in a Spinco, model L, centrifuge. The resultant supernatant fluid was used immediately for the assay of uridine kinase and uridine phosphorylase activity. Freshly prepared cell extracts were always used to avoid loss of enzyme activity. Storage of the extracts at −20° C. for 75 days resulted in the loss of 90% of these activities, and the activities were also lost upon lyophilization of the extracts (D. J. Rainnie and D. G. R. Blair, unpublished observations).

The protein content of the cell-free extract was determined by the method of Lowry *et al.* (1951) with the use of bovine serum albumin (fraction V) as a standard. The haemoglobin content was estimated by the cyanomethaemoglobin method of Evelyn and Malloy (1938) and was subtracted from the total protein content for the purpose of calculating enzyme specific activities. Cells from grossly bloody ascites tumours were not used.

*Assay of enzyme activities*

Uridine kinase and uridine phosphorylase activities of the cell-free extracts of the tumour cells were estimated simultaneously by a radioisotopic method similar to that employed by Sköld (1960*d*) for the measurement of these activities in acetone powder extracts of EAC cells.

The enzyme reaction medium (0·3 ml.) contained $MgCl_2$ (40 mM); tris(hydroxymethyl)aminomethane (Tris) buffer, pH 7·4 (33 mM); phosphate buffer, pH 7·4 (25·5 mM); ATP (13·3 mM); potassium D-3-phosphoglycerate (24·7 mM); uridine-2-$^{14}C$ (26·7 mM, 0·032 $\mu$Ci) and the cell extract. Incubations were carried out at 37–38° C. for 15 minutes, and enzymatic activity was terminated by the addition of 0·03 ml. of ice-cold 4 M perchloric acid. The supernatant solution, obtained by centrifugation, was heated in boiling water for 1 hour to break pyrophosphate and purine-glycosyl bonds. Perchlorate ions were precipitated from the cooled solution as $KClO_4$, by neutralization with cold 4 M KOH, and sedimented by centrifugation.

A 0·05 ml. aliquot of the supernatant solution, obtained by centrifugation, was applied to a strip of Whatman No. 1 filter paper (2 cm. or 1 inch wide) and subjected to descending chromatography in absolute ethanol-saturated $Na_2B_4O_7$—5 M ammonium acetate—0·5 M versene (360 : 160 : 40 : 1, v/v) (Reichard and Sköld, 1958). Standards of uracil, uridine, UMP and adenine were chromatographed at the same time. The ultraviolet (u.v.) light-absorbing substances were located on the developed chromatograms with a " Chromatolite " u.v. lamp, and areas corresponding to uracil, uridine and UMP on standard chromatograms were cut out and immersed in 3·0 ml. of distilled $H_2O$ for at least 24 hours. Aliquots (0·25 ml.) of the eluate were spread on aluminium planchets and dried in air at room temperature, and the radioactivity was determined in a Nuclear-Chicago gas-flow system as described by Blair and Hall (1965). The percentages of total radioactivity in uracil, uridine and UMP were calculated for each sample, and the amounts of uracil and UMP formed from uridine-2-$^{14}C$ were calculated from these values and the specific activity of uridine-2-$^{14}C$.

Because the enzymatic formation of uracil and UMP is not perfectly linear with enzyme concentration (Sköld, 1960*d*), the assay for each cell extract was done with several enzyme concentrations to ensure the rectilinear portion of the activity curve. Fig. 1 shows the formation of uracil and UMP (15 minutes) versus the

Fig. 1.—Dependence of uridine kinase (●——●) and uridine phosphorylase (●– – – –●) activities on the amount of EAC/s cell extract. The reaction time was 15 minutes.

amount of EAC/s cell extract in a representative experiment. Enzyme activities were calculated from the initial slopes of the activity curves (Fig. 1) as μmoles of uracil or UMP formed per mg. of protein (corrected for haemoglobin) per hour. Fig. 2 shows that the formation of UMP was linear with time for 15 minutes, under the conditions described above, while uracil formation was linear for about 30 minutes.

The concentrations of $MgCl_2$ and potassium 3-phosphoglycerate which were used in the enzyme reaction medium were identical to those employed by Sköld (1960*d*), whereas the concentrations of ATP, Tris, phosphate and uridine-2-$^{14}C$ were, respectively, 1·65, 1·15, 1·55 and 15·3 times as large as those used by Sköld (1960*d*). Fig. 3 and 4 show that the concentrations of phosphate and uridine, respectively, which were used in the present experiments were optimal. Adenosine 5′-triphosphate was retained throughout the enzymatic reaction, and its concentration, measured by a combined spectrophotometric and anion-exchange

FIG. 2.—Uridine kinase (●——●) and uridine phosphorylase (●– – – –●) activities of a EAC/s cell extract as a function of reaction time. The volume of cell extract was 0·04 ml.

FIG. 3.—Dependence of the uridine kinase (●——●) and uridine phosphorylase (●– – – –●) activities of a EAC/s cell extract on phosphate concentration. The volume of cell extract was 0·04 ml., and the reaction time was 15 minutes.

resin chromatographic method (Blair, Stone and Potter, 1960), at the end (15 minutes; 0·06 ml. of EAC/s cell extract) was 7·18 mM. When the initial ATP concentration was 8·0 mM rather than 13·3 mM, UMP formation was 98%, and uracil formation 124%, of the formation at the higher concentration.

FIG. 4.—Dependence of the uridine kinase (●——●) and uridine phosphorylase (■– – – –■) activities of a EAC/s cell extract on uridine concentration. The volume of cell extract was 0·04 ml., and the reaction time was 15 minutes.

### RESULTS

*Specific activities of uridine kinase and uridine phosphorylase in extracts of EAC/s cells*

The specific activities of uridine kinase and uridine phosphorylase in extracts of EAC/s cells varied appreciably, and apparently randomly, over the span of 35 cell transplant generations studied (Fig. 5). The mean uridine phosphorylase specific activity varied from a high of 1·413 μmoles/hr/mg. protein at cell transplant generation No. 16 to a low of 0·520 μmole/hr/mg. protein at generation No. 33. The range of values for individual samples was 0·348–1·413 μmoles/hr/mg. protein. The mean uridine kinase specific activity of the same cell extracts varied from 2·610 μmoles/hr/mg. protein at cell transplant generation No. 24 to 0·960 μmole/ hr/mg. protein at generation No. 12. The range of values for individual samples was 0·960–2·880 μmoles/hr/mg. protein.

Cells of transplant generations 12–14 were obtained from 10-day-old tumours, whereas cells of generations 16, 24, 33, 36 and 46 came from 8-day-old tumours, and cells of the other generations were from 9-day-old tumours. Close examination of Fig. 5 shows that there is no obvious correlation between the activities and the age of the tumour: The highest and lowest activities of uridine phosphorylase were obtained for extracts from 8-day-old tumours, and, although the highest uridine kinase activity was obtained for an extract of cells from an 8-day-old tumour, the third-lowest activity was also obtained for cells from a tumour of the same age. However, the average mean specific activities ($\mu$moles/hr/mg. protein $\pm$ standard deviation) of uridine kinase for 8, 9 and 10-day-old tumour cells were 1·932 $\pm$ 0·554, 1·481 $\pm$ 0·416 and 1·632 $\pm$ 0·536, respectively, and the corresponding average mean specific activities for uridine phosphorylase were 0·946 $\pm$ 0·334, 0·831 $\pm$ 0·233 and 0·715 $\pm$ 0·157, respectively. There was no significant difference between the three values in each group.

FIG. 5.—Specific activities of uridine kinase (●——●) and uridine phosphorylase (●- - - -●) in extracts of EAC/s cells at successive transplant generations. The vertical lines indicate ranges of values.

Growth of the EAC/s tumour in male Swiss mice was similar to that described by Klein and Révész (1953) for the Ehrlich ascites carcinoma, with no definite exponential phase, and the terminal phase began at about 9 days after cell inoculation (6·2 $\times$ $10^6$ cells) (Rainnie, 1967; Rainnie and Blair, 1969). The viability of free cells, collected from the peritoneal cavities of 10 tumour-bearing mice each day during the determination of the growth curve, was estimated by a dye-exclusion method (Phillips and Terryberry, 1957) with erythrosin B. The viability of cells ranged from 71·8 to 92·7% during the entire growth period, and was 83·1, 87·1 and 89·4% for cells from 8-, 9- and 10-day-old tumours, respectively (Rainnie, 1967). Cell-size distribution studies (Coulter cell counter and plotter) on the same cell populations did not indicate any change in the constituent populations between days 3 and 12 of tumour growth (Rainnie, 1967; Rainnie and Blair, 1969).

*Specific activities of uridine kinase and uridine phosphorylase in extracts of EAC/AzU$_1$ and EAC/AzU$_2$ cells*

The specific activities of uridine kinase and uridine phosphorylase in extracts of EAC/AzU$_1$ and EAC/AzU$_2$ cells from 8- or 9-day-old tumours also varied considerably over the 33 and 17 cell transplant generations, respectively, which were investigated (Fig. 6). No trend could be observed toward decreased or increased uridine phosphorylase specific activities with 6-azauracil treatment. The mean

FIG. 6.—Specific activities of uridine kinase (●) and uridine phosphorylase (■) in extracts of EAC/AzU$_1$ (——) and EAC/AzU$_2$ (- - - -) cells at successive transplant generations. The vertical bars indicate the range of values.

specific activity of the enzyme from EAC/AzU$_1$ cells varied from 0·304 μmole/hr/mg. protein at cell transplant generation No. 15 to 0·958 μmole/hr/mg. protein at generation No. 12, while that from EAC/AzU$_2$ cells varied from 0·396 μmole/hr/mg. protein at transplant generation No. 17 to 1·125 μmoles/hr/mg. protein at generation No. 12. Remarkable similarity is seen in the specific activities, and in the variation, from transplant generation to transplant generation, in the activities of the enzyme from the two cell sublines. This result suggests (a) that cells of the two sublines are biochemically similar and (b) that the enzymes from both types of cells react similarly to factors involved in assaying the enzyme activity.

The specific activity of uridine kinase from EAC/AzU$_1$ cells appeared first of all to increase, and, later, to decrease with AzU treatment. A similar rise was observed for the EAC/Az$U_2$ subline, but this subline was discontinued before any subsequent decrease was observed. The mean specific activity of uridine kinase from EAC/Az$U_1$ cells varied from 1·210 $\mu$moles/hr/mg. protein at cell transplant generation No. 33 to 2·740 $\mu$moles/hr/mg. protein at generation No. 8, while the specific activity of the enzyme from EAC/Az$U_2$ cells varied from 1·910 $\mu$moles/hr/mg. protein at generation No. 15 to 3·290 $\mu$moles/hr/mg. protein at generation No. 17, when the subline of cells was discontinued. No continuous trend was observed toward increased or decreased enzyme activity with 6-azauracil treatment.

FIG. 7.—Specific activities of uridine kinase (●——●) and uridine phosphorylase (●– – – –●) in extracts of EAC/AzUR cells at successive transplant generations. The vertical bars indicate the range of values.

*Specific activities of uridine kinase and uridine phosphorylase in extracts of EAC/AzUR cells*

The specific activities of the two " uracil pathway " enzymes in extracts of EAC/AzUR cells also varied considerably over 11 cell transplant generations from the commencement of treatment with 6-azauridine, without any definite trend toward increased or decreased activities (Fig. 7). In this case the enzyme specific activities were determined at each successive transplant generation, where possible, in order to ascertain the changes from one generation to the next.

The mean specific activity of uridine phosphorylase varied from 0·540 $\mu$mole/hr/mg. protein at transplant generation No. 9 to 1·625 $\mu$moles/hr/mg. protein at generation No. 6, while the mean specific activity of uridine kinase varied from 0·626 $\mu$mole/hr/mg. protein at generation No. 2 to 1·740 $\mu$moles/hr/mg. protein at generation No. 7.

With the exception of cells of generation No. 9, which were obtained from 8-day-old tumours, EAC/AzUR cells were harvested from 9-day-old tumours.

*Development of resistance of EAC/AzU$_1$ and EAC/AzU$_2$ cells to 6-azauracil and of EAC/AzUR to 6-azauridine*

Although the Ehrlich ascites carcinoma is only mildly sensitive to inhibition by 6-azauracil (Šorm and Keilová, 1958), decreased sensitivity to the drug could be demonstrated, by host survival time, for both the EAC/AzU$_1$ (Table I) and the

TABLE I.—*Development of Resistance to 6-azauracil in the EAC/AzU$_1$ Subline of the Ehrlich Ascites Carcinoma (EAC/s)*

| Cell transplant generation | Dose of 6-azauracil (mg./kg./day for 6 days) | No. of mice T* | C* | Mean survival time in days ± S.D. T* | C* | T.I.† |
|---|---|---|---|---|---|---|
| 1 | 250 | 5 | 5 | 15·8±1·72 | 15·6±1·96 | 0·013 |
| 2 | | 6 | 6 | 15·0±0·58 | 15·7±0·94 | −0·045 |
| 3 | | 5 | 6 | 16·6±3·20 | 15·5±2·69 | 0·071 |
| 4 | 750 | 6 | 6 | 20·0±2·08 | 16·7±1·38 | 0·198 |
| 7 | | 6 | 6 | 17·0±2·00 | 15·0±0·58 | 0·133 |
| 10 | | 5 | 6 | 19·4±2·58 | 18·5±3·30 | 0·049 |
| 12 | | 5 | 5 | 17·6±1·20 | 16·4±1·50 | 0·073 |
| 13 | | 6 | 5 | 16·2±0·93 | 16·2±0·75 | 0 |
| 14 | | 6 | 6 | 17·7±2·06 | 18·8±2·91 | −0·062 |
| 16 | | 5 | 5 | 15·6±1·74 | 17·8±1·72 | −0·124 |
| 18 | | 5 | 5 | 15·4±1·96 | 17·2±2·48 | −0·104 |
| 24 | | 5 | 4 | 18·4±1·20 | 18·3±0·83 | 0 |
| 27 | | 6 | 5 | 17·0±1·41 | 18·0±1·10 | −0·055 |
| 29 | | 6 | 6 | 13·8±1·86 | 14·5±1·39 | −0·048 |
| 33 | | 4 | 5 | 21·0±4·64 | 21·4±4·27 | −0·019 |

* T, treated; C, control.

† T.I., therapeutic index.

EAC/AzU$_2$ (Table II) sublines after about 12 transplant generations under treatment with AzU. The parent EAC/s cells (transplant generation No. 1 in Tables I and II) were not very sensitive to an AzU dose of 250 mg./kg./day (Table I) but were sensitive to a doubled dose (Table II). The effect of changing the dose of AzU from 250 to 750 mg./kg./day for EAC/AzU$_1$ cells at transplant generation No. 4 was an increase in the therapeutic index which later decreased to zero at generation No. 13.

Decreased sensitivity of EAC/AzUR cells to 6-azauridine was apparent after 12 cell transplant generations under treatment with 150 mg. of AzUR/kg./day for three generations and with 300 mg. of AzUR/kg./day for nine generations (Table III). Change of the dosage from 150 to 300 mg./kg./day produced an appreciable increase in the therapeutic index which decreased in succeeding generations.

In Table IV the mean survival times of mice inoculated with EAC/s or EAC/AzU$_1$ cells and treated with AzU, AzUR or FU are presented. The therapeutic index for a dose of 750 mg. of AzU/kg./day against the EAC/s tumour was 0·163 to 0·339, whereas it was 0·032 against the 32nd transplant generation of the EAC/AzU$_1$ tumour. Comparison of the T.I. for 150 mg. of AzUR/kg./day against the EAC/s tumour with that against the EAC/AzU$_1$ tumour shows that the latter was cross-resistant to this dose of azauridine. On the other hand, the EAC/AzU$_1$ tumour was more sensitive than the parental EAC/s tumour to FU.

TABLE II.—*Development of Resistance to 6-azauracil in the EAC/AzU$_2$ Subline of the Ehrlich Ascites Carcinoma (EAC/s)*

| Cell transplant generation | No. of mice T* | No. of mice C* | Mean survival time in days ± S.D. T* | Mean survival time in days ± S.D. C* | T.I.† |
|---|---|---|---|---|---|
| 1 | 5 | 6 | 17·2±2·49 | 15·8±4·26 | 0·202 |
| 3 | 5 | 6 | 19·6±2·42 | 15·7±2·21 | 0·249 |
| 4 | 6 | 6 | 17·0±1·53 | 13·7±1·70 | 0·241 |
| 8 | 5 | 6 | 18·0±1·67 | 15·2±1·57 | 0·184 |
| 10 | 4 | 6 | 19·5±1·12 | 17·0±3·51 | 0·147 |
| 11 | 6 | 6 | 15·7±1·11 | 15·7±1·27 | 0 |
| 12 | 5 | 6 | 15·4±2·06 | 17·0±0·82 | −0·094 |
| 13 | 6 | 6 | 16·8±1·46 | 17·3±0·94 | −0·029 |
| 14 | 6 | 6 | 18·8±0·68 | 19·2±0·69 | −0·018 |
| 15 | 5 | 6 | 18·8±5·76 | 16·5±4·42 | 0·139 |
| 16 | 5 | 6 | 17·0±1·79 | 18·8±1·46 | −0·097 |
| 17 | 4 | 6 | 16·3±1·15 | 14·7±1·25 | 0·108 |

* T, treated; C, control. Treated animals received 500 mg. 6-azauracil/kg./day for 6 days.
†T.I., therapeutic index.

TABLE III.—*Development of Resistance to 6-azauridine in the EAC/AzUR Subline of the Ehrlich Ascites Carcinoma (EAC/s)*

| Cell transplant generation | Dose of 6-azauridine (mg./kg./day for 6 days) | No. of mice T* | No. of mice C* | Mean survival time in days ± S.D. T* | Mean survival time in days ± S.D. C* | T.I.† |
|---|---|---|---|---|---|---|
| 1 | 150 | 6 | 6 | 19·2±5·13 | 15·0±1·53 | 0·280 |
| 2 | | 6 | 5 | 21·5±5·99 | 14·8±5·04 | 0·452 |
| 3 | | 6 | 6 | 18·2±2·97 | 13·6±1·60 | 0·338 |
| 4 | 300 | 6 | 5 | 18·3±4·42 | 9·8±0·98 | 0·870 |
| 7 | | 4 | 6 | 11·0±1·23 | 11·2±1·77 | −0·018 |
| 8 | | 4 | 6 | 16·0±2·24 | 12·0±1·15 | 0·330 |
| 9 | | 4 | 6 | 14·5±0·38 | 17·2±2·14 | −0·157 |
| 10 | | 5 | 6 | 16·0±3·29 | 13·2±1·46 | 0·212 |
| 12 | | 6 | 6 | 17·0±3·17 | 17·5±1·98 | −0·029 |

* T, treated; C, control.

† T.I., therapeutic index.

TABLE IV.—*Effect of 6-azauracil, 6-azauridine and 5-fluorouracil Treatment on the Survival of Mice Inoculated with EAC/s or EAC/AzU$_1$ Ehrlich Ascites Carcinoma Cells*

| Cells | Cell transplant generation | Drug | Dose (mg./kg./day for 6 days) | No. of mice T* | No. of mice C* | Mean survival time in days ± S.D. T* | Mean survival time in days ± S.D. C* | T.I.† |
|---|---|---|---|---|---|---|---|---|
| EAC/s | 29 | AzU | 750 | 6 | 6 | 19·0±2·31 | 16·3±3·20 | 0·163 |
| | 44 | AzU | 750 | 6 | 6 | 25·7±6·10 | 19·2±3·02 | 0·339 |
| | 46 | AzUR | 150 | 6 | 6 | 19·2±5·13 | 15·0±1·53 | 0·280 |
| | 60 | FU | 25 | 6 | 6 | 25·5±4·99 | 19·5±3·20 | 0·308 |
| EAC/AzU$_1$ | 32 | AzU | 750 | 6 | 6 | 18·2±3·45 | 17·7±5·06 | 0·028 |
| | 34 | AzUR | 150 | 6 | 6 | 16·7±4·42 | 17·5±2·06 | −0·046 |
| | 48 | FU | 25 | 6 | 6 | >49·8‡ | 16·8±0·90 | >1·964 |

* T, treated; C, control.
† T.I., therapeutic index.
‡ One mouse alive after 132 days without any gross sign of tumour growth.

Table V presents the mean total packed cell volume data for the experiments with 6-azauracil and 6-azauridine presented in Table IV, and for some additional experiments with EAC/s cells with 6-azauracil and 6-azauridine. It is readily apparent that corresponding doses of AzU or AzUR inhibited the growth of EAC/s cells more effectively than that of EAC/$AzU_1$ cells. Thus, these data support the survival data in showing that the EAC/$AzU_1$ cells are less sensitive to AzU and AzUR than are EAC/s cells.

TABLE V.—*Effect of 6-azauracil and 6-azauridine on the Total Packed Cell Volume of EAC/s and EAC/$AzU_1$ Ehrlich Ascites Tumours*

| Cells | Cell transplant generation | Drug | Dose (mg./kg./day for 6 days) | No. of mice T* | No. of mice C* | Mean TPCV (ml. ± S.D.) T* | Mean TPCV (ml. ± S.D.) C* | T/C |
|---|---|---|---|---|---|---|---|---|
| EAC/s | 44 | AzU | 750 | 6 | 6 | 0·77±0·58 | 2·14±0·39 | 0·36 |
| | 136 | AzU | 750 | 8 | 10 | 0·25±0·23 | 1·05±0·37 | 0·24 |
| | 46 | AzUR | 150 | 6 | 6 | 0·54±0·02 | 1·85±0·08 | 0·29 |
| | 134 | AzUR | 300 | 7 | 9 | 0·29±0·21 | 1·23±0·87 | 0·24 |
| EAC/$AzU_1$ | 32 | AzU | 750 | 5 | 5 | 1·91±0·20 | 2·58±0·23 | 0·74 |
| | 34 | AzUR | 150 | 4 | 6 | 1·01±0·26 | 1·58±0·09 | 0·64 |

* T, treated; C, control.

Comparison of the enzyme specific activities (Fig. 6) of the EAC/$AzU_1$ subline with the azauracil-sensitivity data (Tables I, IV and V) shows that there was no correlation between uridine kinase and uridine phosphorylase activity of the cell extracts and sensitivity to azauracil. Similarly, there was no apparent correlation between the enzyme specific activities of the EAC/$AzU_2$ cells (Fig. 6) and sensitivity to azauracil (Table II), or between the enzyme specific activities of the EAC/AzUR cells (Fig. 7) and sensitivity to azauridine (Table III).

### DISCUSSION

In the experiments described in this report, alterations in the specific activities of uridine kinase or of uridine phosphorylase, which are activating enzymes for azauracil (Pontis, Degerstedt and Reichard, 1961; Sköld, 1960*c*), could not be correlated with the development of resistance to 6-azauracil and 6-azauridine in AzU- and AzUR-treated sublines of the Ehrlich ascites carcinoma. This result is similar to that obtained by Reichard *et al.* (1962) in regard to the development of resistance of Ehrlich ascites carcinoma cells to 5-fluorouracil. In that case two tumour lines became resistant to FU before a decrease in either uridine kinase or uridine phosphorylase occurred, and it appeared that other factors were responsible for the development of the resistant state.

One mechanism of resistance to 6-azauridine, which has been definitely established for L-5178-Y cells cultured *in vitro*, is decreased uridine kinase activity (Pasternak *et al.*, 1961). Our results suggest that this mechanism was not involved in the development of resistance to AzU in EAC/$AzU_1$ and EAC/$AzU_2$ cells, although EAC/$AzU_1$ cells were cross-resistant to AzUR, and in the development of resistance to AzUR in EAC/AzUR cells. The lack of cross-resistance of EAC/$AzU_1$ cells to FU supports this conclusion. Another mechanism (or mechanisms), such as decreased cellular permeability to the drugs, decreased sensitivity of orotidylate decarboxylase to inhibition by azauridylic acid or increased synthesis of pyrimidines *de novo* (Fallon *et al.*, 1962), appears to be operative. The work of Pinsky and Krooth (1967*a*, 1967*b*) on the growth of human diploid cell strains in the presence of AzUR suggests that resistance to AzU and AzUR could involve an increase in the specific activity of the target enzyme, orotidylate decarboxylase, in response to an accumulation of a precursor of UMP.

The decrease in sensitivity to AzU and AzUR which developed in the sublines of the EAC/s tumour was not great, however, and may not have been extensive enough for any significant alterations in the specific activities of uridine kinase and uridine phosphorylase to be detected. The variability of these activities in different transplant generations of the parent EAC/s cells, makes it clear that only relatively large changes would be significant. The specific activity of uridine phosphorylase in extracts from individual 8- to 10-day-old EAC/s tumours varied from 0·348 to 1·413 $\mu$moles/hr/mg. protein over the span of 35 cell generations, while that of uridine kinase varied from 0·962 to 2·880 $\mu$moles/hr/mg. protein, the highest values being 4·1 and 3·0 times the lowest, respectively. In comparison, a similar range of values (50–140 $\mu$moles of UMP or uracil/15 minutes/mg. acetone powder) was reported by Sköld (1960*d*) for activities of these two enzymes in acetone powders prepared from EAC cells from tumours of unstated age and inoculum size. The range of values found by Reichard *et al.* (1962) and Sköld *et al.* (1962) for uridine kinase activity in acetone powders of ELD Ehrlich ascites cells was 40–70 units.

It was pointed out in the " Results " section that the mean activities of either uridine kinase or uridine phosphorylase in extracts from 8-, 9- or 10-day-old tumours were not significantly different with respect to tumour age. Recent experiments (Rainnie and Blair, 1969), in which the specific activities of the two enzymes were measured in separate assays concurrently with the progression of growth of the Ehrlich ascites carcinoma from an inoculum of $5{\cdot}2 \times 10^6$ cells, indicated that the specific activity of uridine phosphorylase was significantly greater in extracts of cells from 10-day-old tumours than in extracts of cells from 8- or 9-day-old tumours. The results reported in this communication differ from those cited above (Rainnie and Blair, 1969), but a direct comparison of the two sets of data is not valid because of (1) the different assay methods and (2) the use of cells from different cell transplant generations, which may have been initiated with different cell doses, thus perhaps shifting the growth curve, in the one case, and of cells from only one cell transplant generation in the other.

Reichard *et al.* (1962) considered the irregular variations which they observed in the activities of uridine phosphorylase, uridine kinase and deoxyuridine phosphorylase in acetone powders from four FU-resistant EAC cell lines to be random and probably caused by the inhomogeneity of the animals which were used. Physiological variation was undoubtedly a factor in those experiments, as well as in those described in this report. Numerous other factors must be involved, however, and it is not possible at this time to state what portion of the total effect is contributed by physiological variation. It is apparent that the use of freshly prepared cell extracts or of acetone powders provides essentially the same degree of variation in enzyme specific activities.

Demonstration of resistance to drug therapy is difficult when the drug is not highly effective against the disease state, as in the case of treatment of the Ehrlich ascites carcinoma with 6-azauracil or 6-azauridine. Simultaneous metabolic changes may be small in such cases, and demonstration of them is more difficult when the range of " normal " values is relatively large, as for uridine kinase and uridine phosphorylase in EAC cells. Small, but nevertheless important, alterations may be completely obscured.

### SUMMARY

The specific activities of uridine phosphorylase and uridine kinase were determined in freshly prepared cell-free extracts of Ehrlich ascites carcinoma cells and of cells from two 6-azauracil-treated sublines and from one 6-azauridine-treated subline over several cell transplant generations for each cell line. All the drug-treated sublines had become less sensitive (more resistant) to the drug with which they were treated after 12 transplant generations under treatment. However, no definite trend toward decreased or increased activities of uridine kinase or uridine phosphorylase could be correlated with the development of resistance. It was found that the specific activities of the two enzymes in all cell lines varied greatly in successive cell transplant generations. This variation may have obscured any changes in the enzyme activities which may have accompanied the development of resistance to 6-azauracil and 6-azauridine. Cells resistant to 6-azauracil were cross-resistant to 6-azauridine but not to 5-fluorouracil.

We thank Mrs. Irene Beck, Mrs. Margaret Dommasch and Mr. L. Rivers for technical assistance, and Hoffman-LaRoche Limited, Montreal, P.Q., Canada, for the generous gift of 5-fluorouracil. This work was supported by the National Cancer Institute of Canada.

### REFERENCES

Blair, D. G. R. and Hall, A. D.—(1965) *Can. J. Biochem.*, **43**, 1857.
Blair, D. G. R., Stone, J. E. and Potter, V. R.—(1960) *J. biol. Chem.*, **235**, 2379.
Chaudhuri, N. K., Montag, B. J. and Heidelberger, C.—(1958) *Cancer Res.*, **18**, 318.
Evelyn, K. A. and Malloy, H. T.—(1938) *J. biol. Chem.*, **126**, 655.
Fallon, H. J., Frei, E. III and Freireich, E. J.—(1962) *Am. J. Med.*, **33**, 526.
Handschumacher, R. E.—(1957) *Biochem. biophys. Acta*, **33**, 428.—(1960) *J. biol. Chem.*, **235**, 2917.
Handschumacher, R. E., Calabresi, P., Welch, A. D., Bono, V., Fallon, H. and Frei, E. III.—(1962) *Cancer Chemother. Rep.*, **21**, 1.
Harbers, E., Chaudhuri, N. K. and Heidelberger, C.—(1959) *J. biol. Chem.*, **234**, 1225.
Klein, G. and Révész, L.—(1953) *J. natn. Cancer Inst.*, **14**, 229.
Lowry, O. H., Rosebrough, N. J., Farr, A. L. and Randall, R. J.—(1951) *J. biol. Chem.*, **193**, 265.
Pasternak, C. A., Fischer, G. A. and Handschumacher, R. E.—(1961) *Cancer Res.*, **21**, 110.
Pasternak, C. A. and Handschumacher, R. E.—(1959) *J. biol. Chem.*, **234**, 2992.
Paterson, A. R. P.—(1960) *Can. J. Biochem. Physiol.*, **38**, 1117.
Phillips, H. J. and Terryberry, J. E.—(1957) *Expl Cell Res.*, **13**, 341.
Pinsky, L. and Krooth, L. S.—(1967*a*) *Proc. natn. Acad. Sci.* U.S.A., **57**, 925.—(1967*b*) *Proc. natn. Acad. Sci.* U.S.A., **57**, 1267.
Pontis, H., Degerstedt, G. and Reichard, P.—(1961) *Biochim. biophys. Acta*, **51**, 138.
Rainnie, D. J.—(1967) M.Sc. Thesis. University of Saskatchewan, Saskatoon, Sask.

RAINNIE, D. J. AND BLAIR, D. G. R.—(1969) *Can. J. Biochem.*, **47**, 429.
REICHARD, P. AND SKÖLD, O.—(1958) *Biochim. biophys. Acta*, **28**, 376.
REICHARD, P., SKÖLD, O. AND KLEIN, G.—(1959) *Nature, Lond.*, **183**, 939.
REICHARD, P., SKÖLD, O., KLEIN, G., RÉVÉSZ, L. AND MAGNUSSON, P.-H.—(1962) *Cancer Res.*, **22**, 235.
SKÖLD, O.—(1960*a*) *Ark. Kemi.* **17**, 51.—(1960*b*) *Ark. Kemi.* **17**, 59.—(1960*c*) *J. biol. Chem.*, **235**, 3273.—(1960*d*) *Biochim. Biophys. Acta.* **44**, 1.
SKÖLD, O., MAGNUSSON, P.-H. AND RÉVÉSZ, L.—(1962) *Cancer Res.*, **22**, 1226.
ŠORM, F. AND KEILOVÁ, H.—(1958) *Experientia*, **14**, 215.